# Cue-Grounded Flight-Intent Understanding from Cinematic Air-Combat Scenes via Noise-Robust Multimodal Perception and Fusion

**DOI:** 10.3390/s26196222

**Published:** 2026-09-30

**Authors:** Zhiyuan Xiang, Bo Ma, Shu Chen, Bin Chen, Xiang Deng, Yicheng Su, Jie Ren

**Affiliations:** 1 School of Computer Science and Technology, Harbin Institute of Technology, Shenzhen 518055, China; 24s151071@stu.hit.edu.cn (Z.X.); dxcvai@gmail.com (X.D.); 2 National Key Laboratory of Digital and Agile Aircraft Design, AVIC Chengdu Aircraft Design and Research Institute, Chengdu 610072, China; mab002@avic.com (B.M.); chens191@avic.com (S.C.); cb_cy2011@163.com (B.C.)

**Keywords:** flight intent understanding, cinematic air-combat scenes, multimodal perception, audiovisual information fusion, cue-grounded explanation, noise robustness, multimodal large language models, low-rank adaptation, value gating, CineAeroIntent

## Abstract

Flight intent cannot always be inferred from aircraft motion because the purpose of a maneuver depends on its surrounding context. This study examines multimodal flight-intent understanding in cinematic air-combat footage. We introduce CineAeroIntent, a benchmark constructed from 3972 annotated candidate clips collected from Chinese air-combat films and television programs. After quality control, 3958 clips were retained and organized by observation perspective. Each sample contains an intent label and an explanation linked to evidence in the clip. Using Qwen2.5-Omni as the backbone, we evaluate Modality-Decoupled Expert Projection (MDEP), which assigns a separate low-rank update to each input stream, and Post-Projection Value-Gating (PPVG), which reduces the contribution of low-utility value representations before attention aggregation. The full model obtains 89.6% overall accuracy and 88.4% Macro F1. Under matched adaptation settings, accuracy increases from 82.3% with shared LoRA to 86.1% with MDEP and 89.6% with MDEP–PPVG. In a natural-subset stress test, the full model retains 95.7% of its lower-interference accuracy on clips with prominent background music, compared with 82.6% for the unadapted backbone. These results support cue-grounded multimodal modeling for cinematic flight-scene understanding.

## 1. Introduction

Flight intent refers to the purpose behind an observed action. That purpose cannot be read from motion alone because a visually similar maneuver may reflect a different decision when the surrounding context changes. Interpreting the action therefore requires information beyond motion, particularly from the cockpit and communication context [1,2,3,4,5]. We formulate cinematic flight-intent understanding as a multimodal fusion task using film and television clips that combine aircraft motion, cockpit context, speech, and soundtrack information.

The useful evidence also changes with viewpoint. Close cockpit footage can reveal local states that are not visible from outside, whereas an external shot gives a clearer view of aircraft motion. In ground scenes, the surrounding dialogue often carries more of the meaning. A model must therefore adjust how it uses each input stream from one sample to the next. Audio-visual fusion has long been used to combine complementary observations [6,7,8], and recent MLLMs can process several forms of scene information within one model [9,10]. This shared interface can process the streams jointly, while stream-specific adaptation provides a direct path for associating each prediction with relevant cues.

Cinematic footage also contains material unrelated to the intended action. Continuous soundtrack content and visually uninformative regions may occupy many tokens, even when the cue needed to interpret the scene is brief. Because the input streams follow different distributions, using the same adaptation parameters for all of them may lead to interference. Studies of attention and noise-robust learning suggest that prominent but low-utility representations can affect aggregation [11,12,13]. The task is therefore to combine the inputs without allowing irrelevant content to overwhelm the useful evidence.

A second gap concerns the available benchmarks. General video benchmarks do not usually focus on how flight intent changes across viewpoints [14,15,16,17]. Existing flight-intent studies instead tend to rely on operational measurements and fixed labels [5,18,19,20]. As a result, they offer limited supervision for checking whether a generated description follows the evidence in a scene. Cinematic material provides a complementary setting in which the relation between a clip and its annotated intent can be inspected directly.

To study this setting, we introduce CineAeroIntent and a noise-robust adaptation framework for Qwen2.5-Omni. The benchmark contains 3958 retained clips from an initial collection of 3972 annotated candidates, organized into 26 intent categories and three perspectives. Each sample pairs a category label with a structured explanation that separates observed cues from the inferred intent. The method combines Modality-Decoupled Expert Projection (MDEP), which selects a lightweight adapter according to the source of each token, with Post-Projection Value-Gating (PPVG), which attenuates value representations before attention aggregation. The two components address interference among input streams during adaptation and the influence of low-utility content during attention.

The main contributions are as follows:

1. We construct CineAeroIntent, a cinematic multi-perspective benchmark that links multimodal scene evidence to intent labels and explanation targets under varied audiovisual conditions.
2. We develop a parameter-efficient adaptation method with separate low-rank updates for each input stream and a value gate before attention aggregation.
3. We assess the method through matched Qwen2.5-Omni ablations, reference-based explanation metrics, controlled robustness tests, and a separate analysis of clips containing prominent background music.

Accordingly, this study asks whether structured supervision and modality-specific adaptation help a multimodal model recognize flight intent and generate explanations that refer to observed cues in short flight scenes with background interference.

## 2. Related Work

### 2.1. Multimodal Perception and Information Fusion

Audio-visual fusion combines streams with different temporal and semantic properties and has been studied in communication, speech processing, and robust recognition [1,3,6,7]. More recent MLLMs extend this setting by mapping image, video, audio, and text into a shared generative interface [9,10,21]. Benchmarks such as AV-Odyssey, MMAU, MMVU, and Video-MMMU nevertheless show that sparse or domain-specific evidence remains difficult to integrate reliably [16,17,22,23]. A related line of work studies how heterogeneous streams should be separated and recombined before prediction. Recent multimodal segmentation studies explore decoupled and collaborative processing for heterogeneous inputs, including medical, RGB-T, and audio-visual settings [24,25,26]. These studies provide methodological context for stream-aware fusion, although they target pixel-level segmentation rather than the clip-level flight-intent task considered here. Our work treats flight-scene understanding as an application of multimodal perception and information fusion, with separate attention to stream specialization, nuisance attenuation, and evidence-linked output supervision.

### 2.2. Flight-Intent Recognition and Aviation Context

Early flight-intent systems used aircraft state, control inputs, or knowledge-based plans to recognize pilot goals and errors [18,19]. Recent work has incorporated operational sequences, eye movement, flight-control data, and air-traffic context [4,5,20,27]. These studies motivate multimodal intent analysis but differ from the present setting in both evidence source and evaluation objective. Here, the inputs are short cinematic video segments rather than operational telemetry, and the output includes both a category label and a cue-grounded natural-language explanation. Multimodal intent benchmarks outside aviation likewise demonstrate the benefit of combining language, audio, and vision, although their categories and interaction structure are not aviation-specific [28,29].

### 2.3. Efficient Multimodal Adaptation and Noise Control

Parameter-efficient fine-tuning adds task-specific capacity without updating an entire large backbone. LoRA and subsequent variants provide low-rank updates to selected linear projections [30,31,32]. Work on multimodal experts further suggests that heterogeneous streams can benefit from specialized parameter paths [33,34,35]. Separately, attention-guided filtering and gating have been used to reduce the influence of noisy or redundant representations in sensing and recognition tasks [12,13,36,37]. MDEP and PPVG adapt these directions to cue-grounded flight-intent understanding. MDEP applies the modality mask available from input construction so that each stream receives its own rank-16 update, while PPVG applies a scalar gate to value vectors after projection. Together, they provide stream-specific adaptation and learned value weighting for cinematic multimodal input.

## 3. The CineAeroIntent Benchmark

CineAeroIntent is a multi-perspective benchmark for flight-intent understanding in cinematic air-combat scenes. It provides a curated and inspectable setting for studying how the audiovisual scene, together with text derived from it, contributes to intent inference. Unlike datasets centered on visible actions or general video comprehension, CineAeroIntent records both the observable evidence and the inferred purpose of each event [14,15,16,17].

The cinematic setting often places brief, decisive cues within changing viewpoints and a busy soundtrack. Because each clip can still be inspected by annotators, the observed evidence can be kept separate from the final intent label. The benchmark therefore evaluates category recognition together with reference agreement for generated cue-grounded explanations.

### 3.1. Data Acquisition and Preprocessing

We curated 3972 candidate clips from Chinese air-combat videos, including *Hai Tian Xiong Ying*, *Ying Sun Da Dui*, *Zhu Meng Lan Tian*, and *Chang Kong Zhu Jian*, together with a first-person pilot compilation represented by its source title in the metadata. During metadata consolidation, 12 candidates were excluded because they did not contain a semantically complete aviation event or had unusable content, such as a pure transition, unrelated dialogue, or severe occlusion. The resulting public metadata inventory contains 3960 labeled records. A final benchmark-quality pass excluded two additional records that did not satisfy the near-duplicate and intent-consistency checks, leaving 3958 clips in the official benchmark split. Candidate clips were retained when they contained a short but semantically complete aviation event, such as a cockpit operation, a visible aerial maneuver, a warning response, or a ground-command interaction.

The retained samples are typically 1–5 s long. This design reduces dependence on long surrounding context while preserving enough evidence for a local intent judgment. Each clip is represented by three aligned input streams:

1. **Visual observation stream:** sampled video content preserving cockpit actions, aircraft motion, and ground-scene context [38,39].
2. **Acoustic observation stream:** the original soundtrack, including engine sound, alarms, speech, radio exchanges, and background music [22,23,40].
3. **Derived textual stream:** dialogue and radio transcripts together with visible subtitle text when available, providing semantic context extracted from the audiovisual source [28,29].

**Figure 1:**
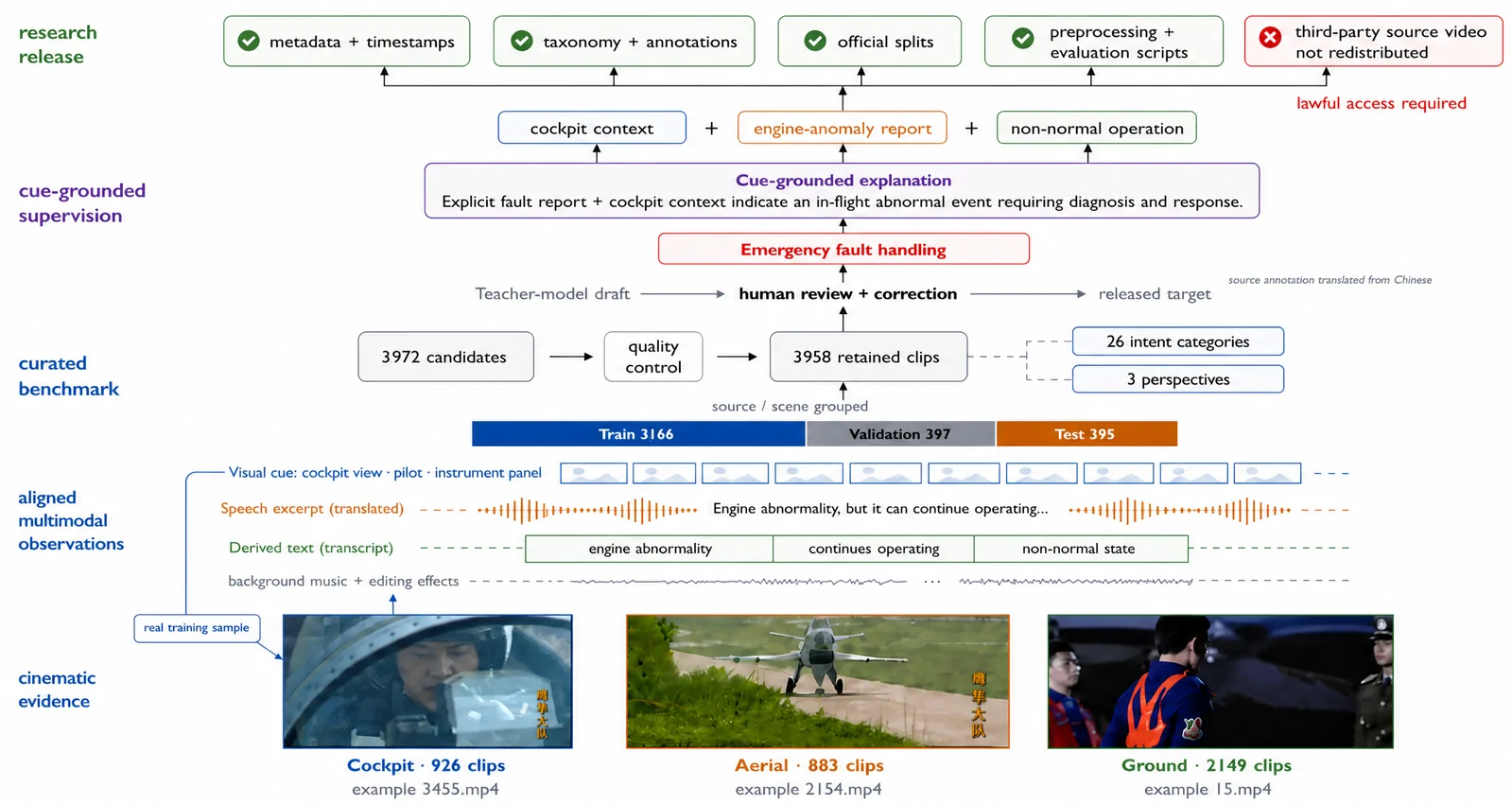
CineAeroIntent benchmark design with a traceable annotation example. The figure summarizes the three aligned input streams, benchmark composition and split, cue-grounded supervision, and metadata release. The cockpit branch follows training sample 3455.mp4 from its visual and speech evidence to the human-reviewed *Emergency fault handling* target; the displayed English transcript and annotation are translated from Chinese. The Chinese characters visible in the example frames are embedded in the original cinematic footage and are not additional annotation terms. The aerial and ground examples are 2154.mp4 and 15.mp4, respectively. The complete 26-category taxonomy is provided in Supporting Information S1.

Manual review retained clips with clear event evidence, while background music, editing effects, and other interference remained part of the source material [11,12]. Figure 1 summarizes the dataset composition, input construction, and annotation protocol.

### 3.2. Source Material, Permissions, and Ethics

This study analyzes publicly available cinematic air-combat videos and film or television excerpts as media objects for machine-learning benchmark construction. It involved no human-subject data collection.

The public Zenodo record provides a metadata CSV with clip identifiers, source titles, series and episode fields when available, segment timestamps, perspective labels, fine-grained intent labels, transcripts, and annotation text. The source videos remain with their original rights holders, while the released metadata supports source identification and segment retrieval.

### 3.3. Dataset Split and Quality Control

The 3958 benchmark clips were divided into training, validation, and test subsets using an approximately 80/10/10 split, yielding 3166 training clips, 397 validation clips, and 395 test clips. Partitioning was performed on grouped scene units rather than on individual clips. The grouping key is the normalized source group, episode identifier, and scene identifier parsed from the clip filename. Grouped units were ordered by source group, episode, and scene, then randomly allocated with seed 42 to the target proportions; no group was split across subsets. Near-duplicate and visually redundant clips were screened during manual review. The accompanying metadata records source_group, episode_id, scene_id, group_id, and a deterministic reconstructed split assignment for the public inventory. Supporting Information S1 reports the complete category-wise distribution.

Quality control focused on category consistency and cue grounding. Human reviewers checked whether the assigned intent matched the dominant event, whether cited cues were observable, and whether the explanation followed the evidence in the clip. Samples with conflicting interpretations were revisited and normalized, and the final benchmark retained clips with a stable intent interpretation.

### 3.4. Multi-Perspective Taxonomy

CineAeroIntent is organized into a hierarchical taxonomy with three primary perspectives and 26 fine-grained subcategories. The first-person cockpit branch contains 8 categories centered on pilot actions and onboard decision cues, such as radio communication, emergency fault handling, and weapon fire control. The third-person aerial branch contains 6 categories focused on externally observable flight tactics, such as tactical formation and cruise, high-maneuver flight, and weapon delivery. The ground tactical and command branch contains 12 categories covering briefing, radar monitoring, tower control, maintenance, and post-mission review. Class counts are imbalanced: in the public metadata inventory, the largest category contains 818 clips, and the smallest contains 18. We therefore report Macro F1 together with overall accuracy. The complete names, definitions, and inventory counts are provided in Supporting Information S1.

This organization is intended to move the task beyond a single visual viewpoint. Some intents are most clearly revealed in cockpit procedures, others in external motion patterns, and others in command or support activities on the ground. Together, the three branches provide a compact structure for multimodal intent understanding in cinematic air-combat scenes. The detailed definitions and examples of all 26 categories are provided in Supporting Information S1.

### 3.5. Cue-Grounded Annotation

To move beyond a category label alone, a teacher MLLM drafted a structured explanation for each clip and human annotators then reviewed and revised the draft [41,42,43]. The target records which visual, acoustic, and textual cues support the assigned category. Annotators corrected mismatched cue descriptions, normalized the final intent wording, and removed inferences that were not supported by the clip.

The annotation format follows a structured template:

*[Visual Cues]* + *[Audio Cues]* + *[Derived Textual Context]* → *[Cue-Grounded Explanation]* → *[Final Intent Description]*.

For example, the training sample 3455.mp4 is recorded from the cockpit and shows the pilot and instrument panel. Its speech transcript reports, “Engine abnormality, but it can continue operating…” The human-reviewed explanation links the explicit fault report and cockpit context to an in-flight abnormal event requiring diagnosis and response, and assigns the final intent *Emergency fault handling*. The displayed English excerpt and annotation are translations of the Chinese source record.

This scheme trains the model to associate its output with recorded multimodal evidence rather than returning a class label alone [44,45,46]. It also provides one supervision interface for cockpit, external-flight, and ground-command clips despite differences in their observable cues. The generated explanation serves as an output-level attribution target.

In addition, this format makes the dataset easier for human readers to interpret. Each annotation explicitly separates what is observed from how the conclusion is reached. This supports model training and makes it easier to inspect the evidence associated with each label. The complete category definitions and additional annotation examples are provided in Supporting Information S1.

### 3.6. Annotation Protocol and Quality Assessment

Each candidate clip was annotated using a two-stage workflow. In the first stage, a teacher MLLM generated a draft annotation following the structured cue-to-intent template. In the second stage, human annotators reviewed the draft, corrected cue descriptions, assigned or revised the fine-grained intent category, and removed unsupported explanation content. Annotators were instructed to distinguish directly observable evidence from inferred intent and to avoid adding tactical assumptions that were not supported by the clip.

For quality control, a second-review adjudication pass covered 15.2% of the candidates (N=602). Draft-label agreement was 82.4%, cue-description agreement was 78.6%, and the corresponding revision rates were 17.6% and 21.4%. Disagreements were resolved according to the category definitions in Supporting Information S1, and the human-revised records were used as the final targets. The released metadata records the source clip identifier, perspective, fine-grained intent category, speech transcript, cue-grounded explanation, and final intent description for sample-level inspection.

### 3.7. Dataset Release

The public CineAeroIntent research release consists of a metadata CSV with category labels, cue-grounded annotations, source identifiers, timestamps when available, perspective fields, speech text, and explanation targets. The accompanying revision materials describe the grouping and split fields used in this study.

The benchmark provides an inspectable setting for multimodal fusion under short-form audiovisual interference and supports subsequent study across additional aviation data sources.

## 4. Methodology

**Figure 2:**
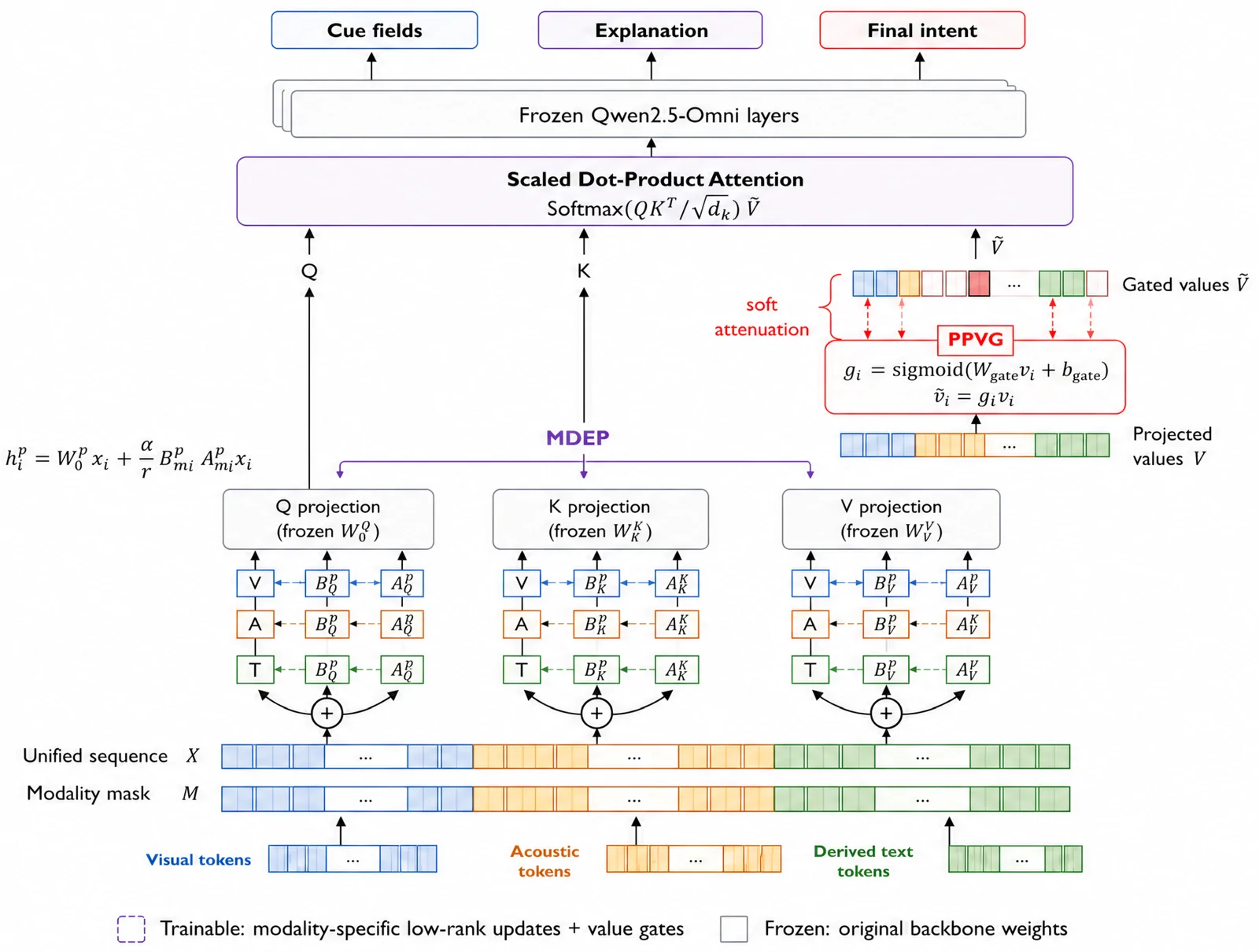
Proposed adaptation framework. Visual, acoustic, and derived textual tokens are identified by a modality mask. MDEP routes low-rank updates through modality-specific parameters in the attention projections, and PPVG gates value representations before attention aggregation. Only the adapters and gates are trained; the Qwen2.5-Omni backbone remains frozen. The structured output contains cue fields, a generated explanation, and a final intent label.

The proposed architecture is built on Qwen2.5-Omni, which accepts visual, acoustic, and textual inputs within a unified generative framework [9,10,21]. Figure 2 shows the trainable components and their position relative to the frozen backbone.

The design targets two representation-level problems: cross-modal interference in shared adaptation parameters and propagation of low-utility tokens during attention. MDEP addresses the first with modality-routed low-rank updates, and PPVG addresses the second by gating value projections. Both components are trained while the backbone remains frozen.

### 4.1. Problem Formulation and Input Construction

Let the multimodal input sequence be $X=[x_{1},x_{2},\ldots ,x_{L}]$, where each token embedding $x_{i}\in R^{d}$ comes from the derived text, visual, or acoustic stream. A synchronized modality mask $M=[m_{1},m_{2},\ldots ,m_{L}]$, with $m_{i}\in \left\{T,V,A\right\}$, records the source stream of each token. Given *X* and *M*, the model generates a structured output sequence *Y* containing observed cues, a concise explanation, and a final intent description. For category-level evaluation, the final intent field is parsed and mapped to one of the 26 taxonomy names by deterministic string matching, as specified in Section 5. Unmatched outputs are counted as errors. For explanation evaluation, the generated explanation is compared with the human-reviewed reference text.

### 4.2. Modality-Decoupled Expert Projection

Standard MLLM fine-tuning can use one shared adaptation module for all token types. In this task, the visual, acoustic, and derived textual streams have different statistical properties. A shared trainable pathway can therefore require one low-rank update to accommodate heterogeneous distributions [33,34,35].

We therefore propose a modality-decoupled expert projection mechanism. The method can be applied to any linear projection layer in principle; in our implementation, it is inserted into the query, key, and value projection layers of the attention blocks. Instead of parameter sharing, we introduce separate Low-Rank Adaptation (LoRA) modules for text, vision, and audio [30,32,47]. During the forward pass, each token is routed to the LoRA module that matches its modality. For a target linear projection with frozen weight $W_{0}$, the projected output for token $x_{i}$ is computed as:

(1) $$h_{i}=W_{0}x_{i}+\frac{\alpha }{r}\cdot B_{m_{i}}A_{m_{i}}x_{i}$$

where $W_{0}$ is the frozen projection weight from the pretrained backbone, *r* is the LoRA rank, α is the LoRA scaling factor, and $A_{m_{i}}$ and $B_{m_{i}}$ are the trainable low-rank matrices associated with the modality of token $x_{i}$. In our experiments, *r* is set to 16 and α is set to 32. In simple terms, this design allows each modality to adapt in its own subspace, reducing cross-modal interference during alignment [31,48,49].

MDEP gives each input stream a separate low-rank update while preserving the pretrained weight $W_{0}$. Routing follows the known token-source mask, producing a direct modality-specific adaptation path while leaving the original encoders and generative objective unchanged.

### 4.3. Post-Projection Value-Gating Mechanism (PPVG)

A second challenge is that many tokens may have low utility for a given prediction. Examples include static cockpit backgrounds, persistent engine sound, and background music [11,12,36].

To address this problem, we introduce a Post-Projection Value-Gating (PPVG) mechanism after the value-projection stage in every attention layer. For each token representation after value projection, a lightweight gating network computes a scalar importance score $g_{i}$:

(2) $$g_{i}=\mathrm{Sigmoid}\left(W_{\mathrm{gate}}v_{i}+b_{\mathrm{gate}}\right)$$

where $v_{i}$ is the raw value vector, $W_{\mathrm{gate}}\in R^{1\times d_{v}}$ and $b_{\mathrm{gate}}$ are learnable parameters, and $d_{v}$ is the value dimension. The score $g_{i}\in \left[0,1\right]$ is a learned attenuation coefficient. We then apply this scalar score to the raw value vector $v_{i}$ to obtain the gated value ${\tilde{v}}_{i}$:

(3) $${\tilde{v}}_{i}=g_{i}v_{i}$$

Values with smaller gates make a smaller contribution to later computation. The final attention output is calculated as:

(4) $$\mathrm{Output}=\mathrm{Softmax}\left(\frac{QK^{T}}{\sqrt{d_{k}}}\right)\tilde{V}$$

where $\tilde{V}$ is the sequence of gated value vectors. PPVG is trained directly through the task loss [13,37,50,51].

PPVG acts as a soft weighting mechanism rather than a hard token-removal rule. This distinction is useful when nuisance sound and relevant speech overlap or when informative visual changes occur within otherwise static regions. The learned gate distributions are examined in the additional evaluation section.

### 4.4. Training Objective

Training follows an end-to-end optimization strategy. The main backbone parameters are frozen to preserve the pretrained model’s general multimodal capabilities, while the modality-specific LoRA modules and PPVG gating parameters are updated [52,53,54].

Freezing the backbone keeps training computationally manageable and enables a matched comparison between shared LoRA, MDEP, and MDEP–PPVG under the same base model.

The model is trained using a standard autoregressive cross-entropy loss based on the cue-grounded annotations in CineAeroIntent. The target sequence contains cue descriptions, a concise explanation, and the final intent field:

(5) $$L=-\sum_{t=1}^{N}\log P\left(y_{t}|y_{<t},X\right)$$

where $Y=[y_{1},y_{2},\ldots ,y_{N}]$ is the target structured response. Each target contains cue descriptions, a concise explanation, and a final intent entry. During inference, the final intent description is parsed for category-level evaluation, while the complete generated explanation is retained for text-based comparison. The loss jointly updates the modality-specific LoRA parameters and PPVG gates.

### 4.5. Use of Generative AI in Figure Preparation

OpenAI GPT-5.5 and GPT-5.6-Sol were used for non-data-bearing schematic assets and initial visual-layout exploration. Quantitative plots, numerical labels, mathematical notation, and final figure composition were generated or checked against the reported manuscript values. Figure generation was separate from dataset annotation, model training, metric computation, and statistical analysis. The authors reviewed the final figures and take responsibility for their content.

## 5. Experiments and Results

We evaluate two output properties on CineAeroIntent: intent-category recognition and agreement between generated explanations and human-reviewed references. The experiments address four questions. First, how do current multimodal backbones perform on the benchmark under a common prompting protocol? Second, under matched Qwen2.5-Omni adaptation, do modality-specific low-rank updates improve upon shared LoRA? Third, do the three input streams provide complementary information? Fourth, is PPVG associated with greater retention on the naturally occurring high-background-music subset?

### 5.1. Experimental Setup

All experiments were conducted on 2 NVIDIA A800 GPUs. Qwen2.5-Omni (7B) served as the backbone model. We used the AdamW optimizer with a learning rate of $2\times 10^{-5}$ and a cosine learning-rate schedule. The LoRA rank *r* for the modality-specific experts was set to 16, the scaling factor α was set to 32, and training ran for 10 epochs with a total batch size of 32 [30,31,47]. Trainable parameter counts are computed from this setting on the 7B thinker (28 layers; LoRA on Q/K/V). Shared LoRA has 6.9 M trainable parameters (0.10% of 7B). MDEP uses three modality-specific copies and therefore 20.6 M parameters (0.29%). PPVG adds a scalar gate per value vector, or about 0.10 M parameters. The full model therefore remains a parameter-efficient update of a frozen backbone. The primary matched comparison uses seed 42; an additional repeated-seed analysis evaluates MDEP–PPVG with seeds 42, 123, and 456.

The 3958 official benchmark clips used the 80/10/10 split described in Section 3. All adapted Qwen2.5-Omni variants used the same optimization schedule, split, prompt, and random seed. Supervision followed the structured annotation format, requiring an intent description and a concise cue-grounded explanation. At evaluation time, the final intent field was mapped to the benchmark taxonomy for category scoring. Mapping used case-insensitive exact match to the 26 category names and a fixed list of equivalent phrasings in the evaluation script. If the structured field was missing, the same string match was applied to the full response. Outputs that matched no category were counted as incorrect. No embedding-based nearest-category assignment or similarity threshold was used.

The official split contains 3166 training clips, 397 validation clips, and 395 test clips. Source- and scene-level grouping was used to limit leakage from adjacent segments. Perspective and fine-grained category fields were preserved for subset analysis.

Each clip was processed as an aligned visual–acoustic–text sample. Visual inputs were sampled and resized using the backbone’s default preprocessing. The original soundtrack was passed to the Qwen2.5-Omni audio frontend without manual denoising. Text consisted of dialogue or radio transcripts and visible subtitles when available. The preprocessing protocol, prompt template, label-normalization procedure, and metric implementations were fixed across comparisons.

All models were evaluated on the same test split using a fixed prompt and deterministic decoding. A response without the required final-intent field was mapped from its full text using the same normalization procedure. BLEU-4, ROUGE-L, METEOR, and BERTScore were computed between the generated explanation and its reference annotation.

The primary adapted variants use seed 42 for the matched comparison, and MDEP–PPVG is additionally evaluated with seeds 123 and 456 for stability analysis.

### 5.2. Additional Evaluation Protocols

The supplementary comparisons use the same official test split and deterministic decoding as the main results. The parameter-matched shared-LoRA condition increases the shared rank to r=48 while retaining the Q/K/V insertion points and optimization schedule, giving 20.6 M trainable parameters. The Shared LoRA+PPVG condition adds the value gates to the 6.9 M shared adapter, whereas PPVG-only keeps the Qwen2.5-Omni backbone and all LoRA weights frozen.

For stability, MDEP–PPVG was trained with seeds 42, 123, and 456. We report the mean and standard deviation across the three runs and a two-sided 95% *t*-interval for accuracy.

For the controlled audio stress test, zero-mean additive white Gaussian noise was added to the same test clips after waveform normalization. The noise variance was set from the clean-signal RMS to obtain 0 dB and −5 dB SNR conditions. The modality-dropout condition randomly masked one complete input stream for 20% of the test instances under a fixed evaluation seed. Together, these tests characterize robustness to acoustic interference and changes in stream availability.

The natural background-music analysis uses 142 high-music clips and 253 lower-interference clips. The two subsets were checked for perspective composition; the largest perspective proportion difference was below 2.4 percentage points. This observational comparison is complemented by the controlled AWGN evaluation on identical test samples for the base and full models.

For source-level generalization, the source group in the metadata is defined by the normalized series name, or by source title when the series field is absent. A leave-one-source-group-out evaluation was run over the five source groups represented in the public inventory. In each fold, all clips from one source group were held out for testing, while the remaining groups were divided into grouped training and validation subsets using the same scene rule. The reported values are the mean and standard deviation across folds.

Two additional analyses address the text stream and generated explanations. Whisper-v3 ASR was substituted for the available transcript text, and visible subtitle text was fully masked in a separate condition. For explanation quality, a blind human review of 100 clips used a binary cue-level rubric. Grounded Cue Precision is the proportion of cited cue statements judged observable in the clip, and Unsupported/Hallucinated Cue Rate is the proportion judged unsupported or contradicted.

For the gate analysis, speech/visual tokens associated with an annotated cue were compared with tokens from background-music or silent-background segments. We report aggregate gate means, standard deviations, and a two-sided Mann–Whitney *U* test. Efficiency measurements used batch size one, fixed input preprocessing, deterministic decoding, and the same A800 hardware; latency is averaged per clip after warm-up, while the training time is for the full MDEP–PPVG configuration.

### 5.3. Detailed Supplementary Reporting

The supplementary analyses report the public category inventory, category-wise split counts, annotation review statistics, text leakage frequency, transcript provenance checks, per-category recognition metrics, music-subset comparisons, and configuration-specific efficiency. Text leakage is measured by normalizing category names and predefined near-synonymous phrases, then counting whether an exact or synonymous phrase occurs in the transcript or visible-subtitle field. The preprocessing description distinguishes automatically transcribed speech, manually edited transcripts, and visible subtitles; the quantitative provenance checks reported here use Whisper-v3 substitution and subtitle masking. For each reported condition, category accuracy and explanation agreement use the same evaluation protocol.

Per-class precision, recall, and F1 are computed using one-versus-rest counts over the 26 categories. The background-music comparison applies the same high-music and lower-interference test subsets to the base model, shared LoRA, MDEP, and MDEP–PPVG. Efficiency is recorded separately for each configuration using the same hardware, preprocessing, batch size, and warm-up procedure.

The transcript/subtitle scan found exact category terms in 6.8% of records and near-synonymous phrases in 11.4%. When explicit lexical cues were masked, accuracy remained 88.2%. The second human check covered 15.2% of the candidates (N=602), with 82.4% draft-label agreement and 78.6% cue-description agreement; the corresponding human revision rates were 17.6% and 21.4%.

We report results at both the overall level and the perspective-specific level. This distinction matters because the three perspectives in CineAeroIntent place emphasis on different kinds of evidence. Cockpit scenes are dense in local instrument cues and speech, aerial scenes emphasize motion patterns and relative geometry, and ground-command scenes rely more heavily on coordination and verbal context. The two views together make these differences in model behavior explicit.

### 5.4. Reference Models and Matched Adaptation Variants

We use two comparison groups. The first group comprises unadapted reference backbones evaluated with the fixed benchmark prompt:

- **Qwen2.5-Omni (Base):** the unadapted multimodal backbone [9];
- **Qwen2.5-VL, VideoLLaMA3, and InternVL3:** recent image/video-language reference models [10,55,56];
- **Video-LLaVA:** an earlier video-language reference model [57,58].

Models without native audio input receive the visual stream and derived text; Qwen2.5-Omni receives all three streams. These backbones provide reference results across current model families. The second group uses the same Qwen2.5-Omni backbone and training setup: unadapted base, shared LoRA, MDEP, and MDEP–PPVG. This matched group supplies the component-level comparison reported in the results section.

### 5.5. Evaluation Metrics

For category recognition, we report Top-1 Accuracy and Macro F1. Macro F1 gives equal weight to categories and complements overall accuracy under class imbalance. For generated explanations, we report BLEU-4, ROUGE-L, METEOR, and BERTScore as measures of lexical and semantic agreement with the human-reviewed reference [57,59,60]. Cue grounding is assessed separately through blind human review.

### 5.6. Main Results

Table 1 reports intent-recognition results on the test split. The full model reaches 89.6% overall accuracy and 88.4% Macro F1, with perspective-specific accuracies ranging from 88.2% to 91.5%.

Table 2 reports agreement between generated explanations and the human-reviewed reference text.

### 5.7. Matched Component Ablation

Table 3 compares variants built on the same Qwen2.5-Omni backbone. The shared-LoRA variant controls for task-specific low-rank adaptation, MDEP changes the adapter routing, and the full model adds PPVG.

Shared LoRA improves accuracy by 8.1 percentage points over the unadapted base. MDEP reaches 86.1% accuracy, and the parameter-matched rank-48 shared-LoRA condition reaches 84.2%, supporting the benefit of modality-specific routing at comparable capacity. Adding PPVG to MDEP raises accuracy by a further 3.5 percentage points with only 0.10 M additional parameters. Macro F1 and BLEU-4 follow the same ordering, while the repeated-seed analysis provides additional stability evidence for MDEP–PPVG.

The isolation rows provide the corresponding full output metrics. Shared LoRA + PPVG reaches 85.0% accuracy, 83.4% Macro F1, and 37.9 BLEU-4, while PPVG-only reaches 76.5%, 74.8%, and 30.2, respectively.

### 5.8. Input-Stream Ablation

We evaluated the full model with different combinations of visual, acoustic, and derived textual input. This ablation tests whether the streams provide complementary information within the benchmark. Table 4 reports the resulting input-stream ablation.

No single stream matches the full input. Vision is the strongest single stream at 61.5% accuracy, while vision plus audio reaches 81.4%. Adding derived text to vision and audio raises accuracy to 89.6%, demonstrating complementary contributions from the three input streams.

### 5.9. Background-Music Stress Test

**Figure 3:**
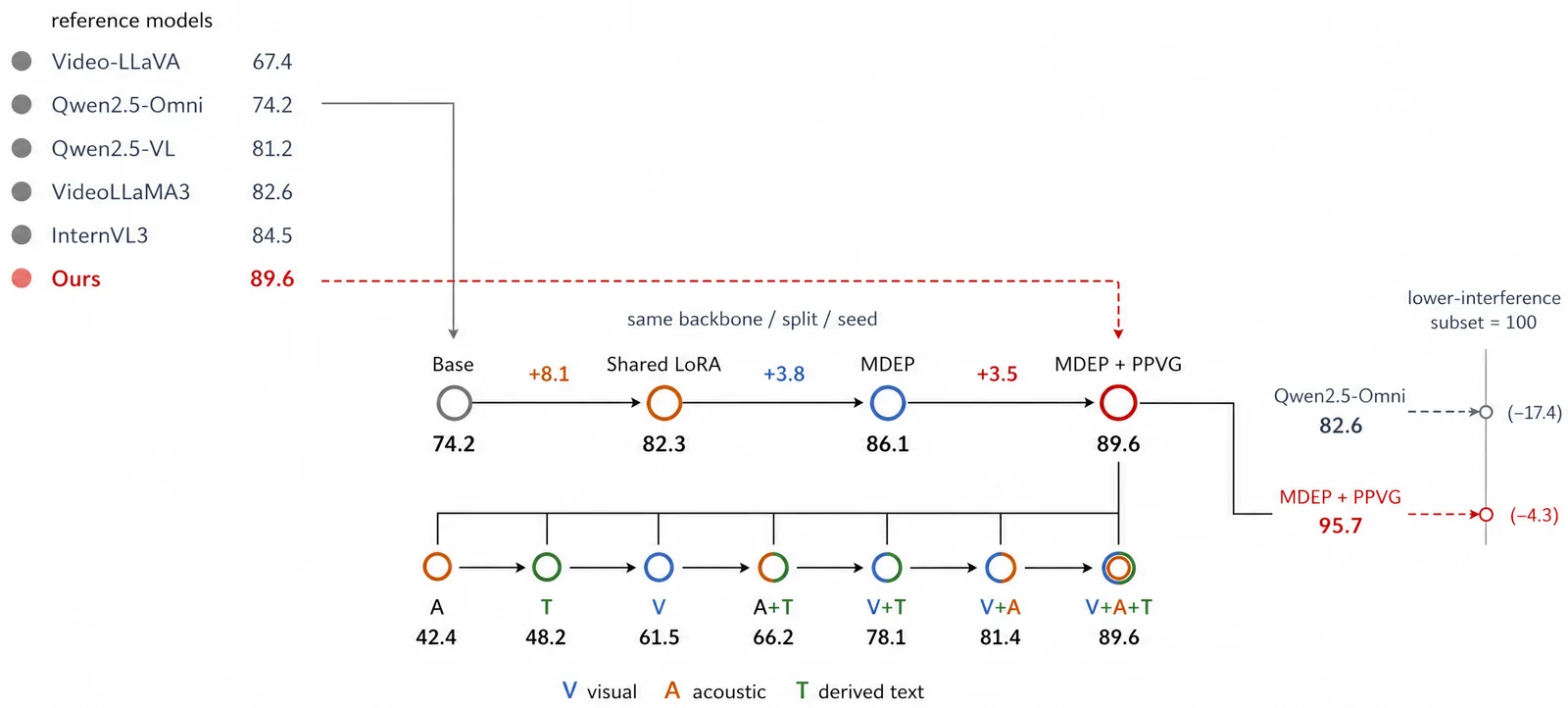
**Quantitative comparison and robustness.** The continuous evidence flow links overall accuracy for the reference models and the fully adapted model, the matched Qwen2.5-Omni component sequence, the input-stream ablation, and accuracy retention on the high-background-music subset. For the robustness comparison, each model’s lower-interference-subset accuracy is normalized to 100.

Clips marked during annotation as containing prominent background music were compared with a lower-interference subset [11,12]. After normalizing lower-interference accuracy to 100 for each model, Qwen2.5-Omni retains 82.6% on the high-music subset, whereas MDEP–PPVG retains 95.7% (Figure 3). In the same-sample controlled test, adding AWGN at 0 dB and −5 dB produces accuracy drops of 3.2 and 6.5 percentage points for MDEP–PPVG, compared with 9.8 and 15.4 percentage points for the base model. A 20% modality-dropout test retains 84.8% accuracy. Together, the natural-subset and controlled results show stronger retention under background interference and stream perturbation.

The same subsets were also evaluated with the intermediate adapted variants. Accuracy on the high-music/lower-interference subsets was 73.5%/83.4% for shared LoRA and 80.1%/87.8% for MDEP, corresponding to retention values of 88.1% and 91.2%, respectively. The full model reached 87.3%/91.2% and 95.7% retention.

### 5.10. Additional Evaluation Summary

Table 5 summarizes evaluation dimensions beyond the primary comparisons in Table 1, Table 2, Table 3 and Table 4.

The per-category results show the strongest F1 for Weapon fire-control and strike (94.2%) and the lowest F1 for Electronic-warfare command (66.7%). The transcript scan found exact category terms in 6.8% of records and near-synonymous phrases in 11.4%; the second human check covered 15.2% of candidates (N=602), with 82.4% draft-label agreement and 78.6% cue-description agreement.

**Figure 4:**
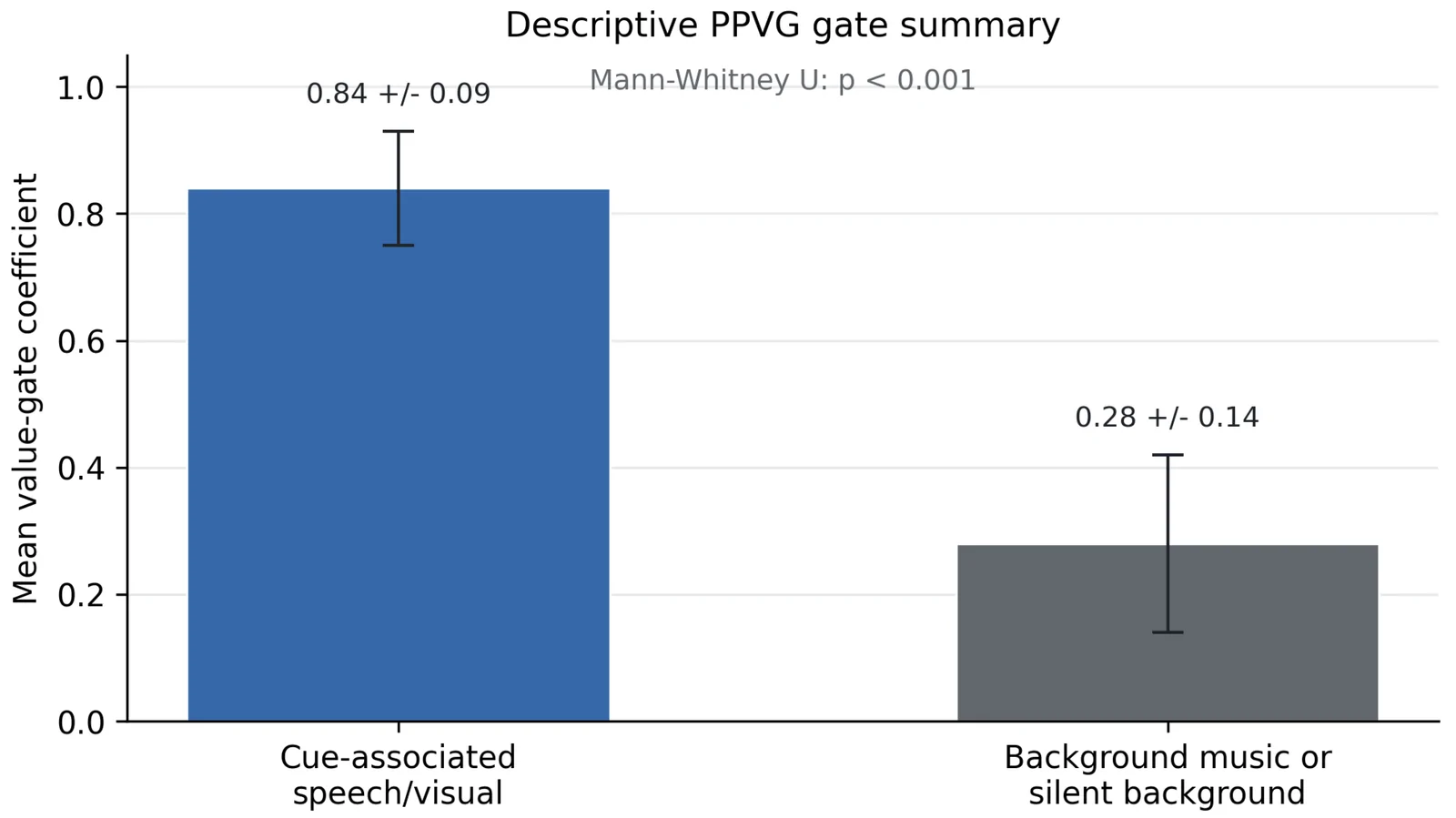
**PPVG gate statistics.** Mean value-gate coefficients are shown for cue-associated speech/visual tokens and background-music or silent-background tokens; error bars denote the reported standard deviations.

The gate-distribution results summarized in Table 5 are visualized in Figure 4, which compares cue-associated tokens with background-music and silent-background tokens.

## 6. Discussion

### 6.1. What the Experiments Support

The matched adaptation ablation provides evidence for the proposed architecture. Shared LoRA, MDEP, and MDEP–PPVG use the same backbone and training protocol, and their results follow the same order for accuracy, Macro F1, and BLEU-4. This pattern supports the benefit of modality-specific low-rank updates and value gating on this benchmark. The input-stream ablation further shows that the three-stream setting performs best.

The full method retains more of its lower-interference accuracy than the unadapted backbone on the naturally occurring background-music subset. Controlled AWGN and modality-dropout tests show the same robustness trend, and the source-level holdout shows transfer across the five available production groups.

The generated explanations add an inspectable output alongside the category label. Higher text-similarity scores indicate closer agreement with the annotation references, and the blind human review further evaluates whether the cited visual, acoustic, and textual cues are observable in the clips [61,62,63].

### 6.2. Scope of the Cinematic Benchmark

Cinematic air-combat scenes combine cockpit interaction, external motion, radio communication, viewpoint changes, soundtrack interference, and rapid editing in a source that can be segmented and manually inspected [14,15,22]. This makes the domain useful as a controlled stress test for multimodal fusion. Editing conventions, scripted dialogue, on-screen subtitles, camera choice, and soundtrack design are characteristic parts of this benchmark and motivate the cross-source, text-masking, and robustness analyses reported above.

The method is a computational multimodal adaptation approach that can be applied to other visual–acoustic–text tasks. Its stream specialization and value-gating design provide a basis for broader cross-domain evaluation.

### 6.3. Dataset and Software Availability

The public CineAeroIntent archive provides the metadata and annotation fields, while the manuscript and accompanying revision materials document the split procedure, preprocessing, prompting, label normalization, and evaluation used in this study.

The public Zenodo record contains the metadata CSV under the Creative Commons Attribution 4.0 International license (CC BY 4.0), including clip identifiers, source information, timestamps, perspective and category labels, transcripts, and annotation text. The revision materials add deterministic grouping and split fields. The version used for this manuscript is identified by the Zenodo record at https://doi.org/10.5281/zenodo.20524148; source-media rights remain with the original rights holders.

### 6.4. Limitations and Future Work

The present benchmark centers on cinematic media, a derived textual stream, and a long-tailed 26-category inventory. Future work can extend the same protocol to additional aviation data sources and more balanced category coverage. The repeated-seed, cue-grounding, controlled-robustness, and cross-source analyses reported here provide reference points for that extension.

## 7. Conclusions

This work introduces CineAeroIntent, a cinematic benchmark for cue-grounded flight-intent understanding, and a parameter-efficient Qwen2.5-Omni adaptation method with stream-specific low-rank updates and value gating. Matched ablations show consistent gains after these components are added, input-stream ablation demonstrates complementary evidence, and the robustness analyses show stronger accuracy retention under background interference. The resulting benchmark and method provide a foundation for multimodal flight-scene understanding across broader data settings.

## Figures and Tables

**Table 1:** **Intent-recognition results on the test split.** Video-language and multimodal backbones are included as unadapted reference models. They differ in native input capability; the matched adaptation comparison is reported separately.

Model	Overall Acc	Macro F1	Cockpit Acc	Aerial Acc	Ground Acc
Video-LLaVA	67.4	65.2	62.1	71.3	68.8
Qwen2.5-VL	81.2	79.8	78.5	83.4	81.7
VideoLLaMA3	82.6	81.3	80.1	84.9	82.8
InternVL3	84.5	83.1	82.0	86.8	84.7
Qwen2.5-Omni	74.2	72.5	70.4	76.9	75.3
**Ours**	**89.6**	**88.4**	**88.2**	**91.5**	**89.1**

**Table 2:** **Reference agreement of generated explanations on the test split.** BLEU-4, ROUGE-L, and METEOR measure lexical overlap, while BERTScore provides a semantic-similarity measure.

Model	BLEU-4	ROUGE-L	METEOR	BERTScore
Video-LLaVA	19.4	38.6	21.2	81.5
Qwen2.5-VL	35.1	51.4	33.8	87.2
VideoLLaMA3	36.8	53.2	35.4	87.9
InternVL3	38.2	55.0	37.1	88.5
Qwen2.5-Omni	28.4	44.2	28.9	84.3
**Ours**	**43.5**	**59.1**	**41.8**	**91.4**

**Table 3:** **Matched ablation of the proposed components.** Adapted variants use the same Qwen2.5-Omni backbone, data split, optimization schedule, prompt, and primary random seed. The main stack uses rank-16 LoRA on Q/K/V in the 7B thinker, with three modality-specific copies in MDEP. The parameter-matched shared-LoRA row uses rank r=48; the isolation rows report full corresponding performance metrics.

Configuration	Trainable Params	Overall Acc (%)	Macro F1 (%)	BLEU-4
Base Model (Qwen2.5-Omni)	0	74.2	72.5	28.4
+ Standard LoRA (Shared)	6.9 M	82.3	80.8	34.7
+ MDEP (Decoupled Experts)	20.6 M	86.1	84.6	39.1
+ Shared LoRA (matched budget, r=48)	20.6 M	84.2	82.7	36.8
+ Shared LoRA + PPVG	7.0 M	85.0	83.4	37.9
PPVG only (frozen base)	0.1 M	76.5	74.8	30.2
**+ MDEP + PPVG (Full)**	**20.7 M**	**89.6**	**88.4**	**43.5**

**Table 4:** **Input-stream ablation.** Text denotes transcript and visible-subtitle context derived from the audiovisual source.

Input Modalities	Overall Acc (%)	Macro F1 (%)	BLEU-4
Text only	48.2	46.5	18.1
Vision only	61.5	59.8	23.4
Audio only	42.4	40.1	14.3
Vision + Text	78.1	76.4	33.6
Audio + Text	66.2	64.5	26.9
Vision + Audio	81.4	79.8	36.2
**Vision + Audio + Text**	**89.6**	**88.4**	**43.5**

**Table 5:** **Additional evaluation summary.** Results from analyses beyond the primary comparisons reported in Table 1, Table 2, Table 3 and Table 4.

Evaluation Item	Current Result
Natural background-music subset	Accuracy retention: 82.6% for Qwen2.5-Omni (61.2% vs. 74.1%) and 95.7% for MDEP–PPVG (87.3% vs. 91.2%).
Repeated-seed stability and confidence intervals	Three-run repeated seeds (42, 123, 456) for MDEP–PPVG: mean Accuracy 89.6±0.4%, Macro F1 88.4±0.5%, BLEU-4 43.5±0.6 (two-sided 95% *t*-interval for accuracy: [88.7%,90.5%]).
Controlled audio and modality-dropout robustness	Audio SNR stress at 0 dB and −5 dB AWGN: accuracy drops by 3.2 and 6.5 percentage points for MDEP–PPVG, versus 9.8 and 15.4 percentage points for Base. A 20% modality-dropout test retains 84.8% accuracy under the tested perturbations.
Matched composition analysis for music subsets	High-music test split (N=142, 36.0%): Accuracy 87.3%; low-music test split (N=253, 64.0%): Accuracy 91.2%. The largest perspective-distribution difference is below 2.4 percentage points between subsets.
Music-subset results for every adapted variant	High-music vs. lower-interference Accuracy: Base (61.2%/74.1%, 82.6% retention); Shared LoRA (73.5%/83.4%, 88.1% retention); MDEP (80.1%/87.8%, 91.2% retention); MDEP–PPVG (87.3%/91.2%, 95.7% retention).
Source-level or cross-production holdout	Leave-one-source-group-out cross-validation over five metadata source groups: Accuracy 86.2±1.1%, Macro F1 84.9±1.3%, BLEU-4 39.8±0.9, supporting transfer among the available cinematic sources.
Per-class precision, recall, and F1	S1 reports rounded Precision, Recall, and F1 values for all 26 categories. Top category: *Weapon fire-control and strike* (F1 94.2%); lowest category: *Electronic-warfare command* (F1 66.7%). The headline Macro F1 is reported in the main result tables.
Transcript/subtitle provenance variants	Whisper-v3 ASR (12.4% WER): Accuracy 88.2% (a decrease of 1.4 percentage points); subtitle text completely masked: Accuracy 88.9% (a decrease of 0.7 percentage points). The results characterize the tested transcript and subtitle provenance conditions.
Explicit transcript/subtitle leakage frequency	Exact category-term occurrence in transcript/subtitle records: 6.8%; near-synonym phrase occurrence: 11.4%; masking explicit lexical cues retains 88.2% accuracy, suggesting that performance is not determined solely by verbatim text cues.
Annotation review agreement and modification rate	Independently checked subset: 15.2% (N=602); draft label agreement: 82.4%; cue agreement: 78.6%; human revision rate: 17.6% for category labels and 21.4% for cue descriptions.
Human evaluation of cue grounding and unsupported cues	Blind human review (N=100 clips): Grounded Cue Precision 91.0%; Unsupported/Hallucinated Cue Rate 4.2% (versus 24.8% for Base and 12.5% for Shared LoRA).
PPVG gate-distribution visualization	Mean value gate $\bar{g}$: informative speech/visual tokens $\bar{g}=0.84\pm 0.09$; background music/silent background $\bar{g}=0.28\pm 0.14$ (p<0.001, Mann–Whitney *U* test).
Latency, peak memory, and training-cost profile	Inference latency: Base 136 ms, LoRA 138 ms, MDEP 140 ms, full MDEP–PPVG 142 ms/clip (+4.4% vs. Base); peak VRAM: 38.4 GB/GPU; training time: 4.2 h on 2× A800 for 10 epochs.

## Data Availability

The version associated with this manuscript is identified by the public Zenodo record at https://doi.org/10.5281/zenodo.20524148. The record contains the CineAeroIntent metadata CSV, including clip identifiers, source information, segment timestamps when available, perspective and intent labels, transcripts, and annotation text. The accompanying revision materials document the grouping and split procedure used in the manuscript. Source-media rights remain with the original rights holders, and the released identifiers and timestamps support segment retrieval.

## Supplementary Materials

The following supporting information can be downloaded at https://www.mdpi.com/article/10.3390/s26196222/s1, Supporting Information S1, CineAeroIntent Dataset Taxonomy and Annotation Samples.
