# Supplementary material for: Cue-Grounded Flight-Intent Understanding from Cinematic Air-Combat Scenes via Noise-Robust Multimodal Perception and Fusion
Source: Sensors (Basel). 2026 Sep 30;26(19):6222. doi: 10.3390/s26196222 (PMC13667636; doi:10.3390/s26196222)
Supplement: Supplementary file 1 [file sensors-26-06222-s001.zip › Supporting_Information_S1.pdf]

Supporting Information S1

CineAeroIntent Taxonomy and Cue-Grounded Annotation Samples

This supporting information provides the CineAeroIntent taxonomy and cue-grounded annotation protocol used for benchmark construction and evaluation.

Taxonomy Overview

CineAeroIntent contains 26 fine-grained intent categories organized into three primary perspectives: first-person cockpit scenes, third-person aerial scenes, and ground tactical or command scenes. Of the 3972 initial candidates, 12 were excluded during metadata consolidation because they did not contain a semantically complete aviation event or had unusable content, producing the public inventory of 3960 labeled records. A final benchmark-quality pass excluded two additional records under the near-duplicate and intent-consistency checks, leaving 3958 clips (cockpit 926, aerial 883, ground 2149) in the official benchmark and a source- and scene-grouped train/validation/test split of 3166/397/395. The largest class contains 818 clips and the smallest contains 18; Macro F1 is therefore reported alongside overall accuracy. The public inventory contains five source groups, with records lacking a series name normalized to the first-person pilot compilation; Table S2 reports these counts. The accompanying metadata version records the source, episode, scene, group, and deterministic seed-42 split fields used to reproduce the grouping protocol on the public inventory.

Table S1: CineAeroIntent category inventory. Counts are from the public metadata release ($n = 3960$).

**Perspective**	**Category**	n	**%**
Cockpit	Radio communication and report	246	6.2
	Tactical maneuver and G-load	169	4.3
	Pre-flight inspection and preparation	151	3.8
	Route cruise and formation	125	3.2
	Emergency fault handling	71	1.8
	Approach, landing, and carrier recovery	65	1.6
	Weapon fire-control and strike	64	1.6
	Ground taxi and takeoff	36	0.9
Aerial	Tactical formation and cruise	298	7.5
	High-maneuver flight and penetration	184	4.6
	Takeoff and climb	164	4.1
	Approach, landing, and arresting recovery	128	3.2
	Weapon delivery and defensive countermeasure	88	2.2
	Helicopter landing and special operations	21	0.5
Ground	Tactical briefing and deployment	818	20.7
	Radar situation monitoring	310	7.8
	Tower takeoff and landing control	271	6.8
	Pre-flight servicing and boarding	232	5.9
	Tactical debrief and performance review	105	2.7
	Carrier-deck special support	93	2.3
	Readiness-level elevation and assembly	86	2.2
	Weapon loading and maintenance	77	1.9
	Special-situation handling and emergency intervention	68	1.7
	Weather assessment and decision	38	1.0
	Ground taxi and marshalling	34	0.9
	Electronic-warfare command	18	0.5
Total		3960	100.0

Table S2: Source groups in the public metadata inventory. Records without a series name are normalized to the first-person pilot compilation group.

Source group	Clips
Hai Tian Xiong Ying	1621
Ying Sun Da Dui	1493
Chang Kong Zhu Jian	578
First-person pilot compilation	258
Zhu Meng Lan Tian	10
Total	3960

Reproducible Metadata Split

The revision materials include machine-readable source, episode, scene, group, seed, and split fields. These fields follow the source/episode/scene grouping rule described in the manuscript and support deterministic reconstruction of the grouped split.

First-person cockpit perspective

This branch covers clips in which the main evidence appears inside the cockpit, including instrument states, pilot actions, warning indicators, speech, and immediate onboard decisions.

1. Radio communication and report: the pilot reports status or responds over radio.
2. Tactical maneuver and G-load: cockpit cues indicate a high-load or tactical maneuver.
3. Pre-flight inspection and preparation: the pilot performs cockpit checks before flight.
4. Route cruise and formation: the cockpit scene indicates cruise, heading-keeping, or formation flying.
5. Emergency fault handling: the pilot responds to a malfunction or abnormal indication.
6. Approach, landing, and carrier recovery: the cockpit scene concerns approach, landing, or recovery.
7. Weapon fire-control and strike: the pilot operates targeting or weapon-related controls.
8. Ground taxi and takeoff: the cockpit scene concerns taxi or takeoff.

Third-person aerial perspective

This branch covers clips in which the main evidence appears through externally visible aircraft motion, formation geometry, engagement posture, or aerial scene structure.

1. Tactical formation and cruise: aircraft hold formation or cruise geometry.
2. High-maneuver flight and penetration: externally visible aggressive maneuvering or penetration.
3. Takeoff and climb: aircraft leave the ground or climb out.
4. Approach, landing, and arresting recovery: externally visible approach, landing, or recovery.
5. Weapon delivery and defensive countermeasure: attack, weapon release, or defensive action.
6. Helicopter landing and special operations: helicopter insertion, landing, or related special-ops motion.

Ground tactical and command perspective

This branch covers clips in which the main evidence appears through ground crew, command posts, support teams, briefings, monitoring stations, or post-mission analysis.

1. Tactical briefing and deployment: personnel brief, assign, or deploy before a mission.
2. Radar situation monitoring: ground personnel monitor radar or related situation displays.
3. Tower takeoff and landing control: tower or control-point instructions for takeoff or landing.
4. Pre-flight servicing and boarding: ground servicing, boarding, or launch preparation around the aircraft.
5. Tactical debrief and performance review: post-event review of performance or tactics.
6. Carrier-deck special support: deck handling or other carrier-specific support.
7. Readiness-level elevation and assembly: personnel assemble or raise readiness.
8. Weapon loading and maintenance: loading, inspection, or maintenance of aircraft or stores.
9. Special-situation handling and emergency intervention: ground response to an abnormal or urgent event.
10. Weather assessment and decision: weather information is used in a ground decision.
11. Ground taxi and marshalling: marshalling or taxi guidance on the ground.
12. Electronic-warfare command: ground command associated with electronic-warfare activity.

Annotation Template

Each sample is annotated using the following structured format:

> [Visual Cues] + [Audio Cues] + [Derived Textual Context] → [Cue-Grounded Explanation] → [Final Intent Description]

Annotators are instructed to record only cues that are observable in the clip. The cue-grounded explanation should state how the observed cues support the final intent without introducing unsupported background assumptions.

Example Annotations

Example 1: Emergency handling

Visual cues: A cockpit warning indicator is active, and the pilot's hand moves quickly toward the control panel.

Audio cues: The audio contains an alarm-like signal and urgent cockpit speech.

Textual context: The transcript indicates a possible engine or system abnormality.

Cue-grounded explanation: The warning display, urgent pilot action, and abnormality-related dialogue support an emergency-response interpretation.

Final intent description: The pilot is performing an emergency handling procedure.

Example 2: Combat engagement

Visual cues: The aircraft maintains an aggressive pursuit posture, and the scene shows target-tracking or weapon-related visual evidence.

Audio cues: The soundtrack includes engine noise and brief communication associated with engagement.

Textual context: The transcript refers to target contact or attack preparation.

Cue-grounded explanation: The visual pursuit pattern and target-related dialogue support an engagement interpretation rather than a generic maneuver label.

Final intent description: The aircraft is executing a combat engagement intent.

Example 3: Command issuance

Visual cues: Ground personnel are shown in a command or monitoring environment.

Audio cues: Voices are directed toward mission coordination or operational response.

Textual context: The transcript contains an instruction or order.

Cue-grounded explanation: The command setting and instruction-like language indicate that the ground team is directing a flight or mission.

Final intent description: Ground command personnel are issuing an operational instruction.

Annotation Reliability and Label Mapping

Teacher-MLLM drafts were revised by human annotators. A second human check covered 15.2% of the candidates ($n = 602$) and was used to correct labels and unsupported explanations. Agreement with the draft was 82.4% for intent labels and 78.6% for cue descriptions; human revision rates were 17.6% and 21.4%, respectively. This second check is treated as an adjudication step within the quality-control workflow. Category evaluation uses case-insensitive string matching to the names above and a fixed list of equivalent phrasings; unmatched outputs are counted as errors. The fixed mapping used for the manuscript evaluation defines the accepted equivalent phrasings.

Supplementary Evaluation Notes

The matched component comparison uses the same Qwen2.5-Omni backbone, source/scene-grouped split, prompt, optimization schedule, and primary seed. The parameter-matched shared-LoRA condition uses rank $r = 48$ and 20.6M trainable parameters. Shared LoRA+PPVG uses the 6.9M shared adapter plus the value gates, while PPVG-only keeps the backbone frozen. MDEP–PPVG was additionally evaluated with seeds 42, 123, and 456; the reported confidence interval is a two-sided t-interval over these three runs.

The controlled robustness results use additive white Gaussian noise at 0 dB and -5 dB SNR on the same test clips, together with a 20% complete-stream dropout condition. The high-background-music comparison is a naturally occurring subset analysis, with perspective proportions differing by less than 2.4 percentage points. The leave-one-source-group-out result uses the five source groups in Table S2 to evaluate transfer across cinematic sources.

On the high-music/lower-interference subsets, accuracy is 61.2%/74.1% for the base, 73.5%/83.4% for shared LoRA, 80.1%/87.8% for MDEP, and 87.3%/91.2% for MDEP–PPVG. The corresponding retention values are 82.6%, 88.1%, 91.2%, and 95.7%. Inference latency is 136, 138, 140, and 142 ms/clip for the base, shared-LoRA, MDEP, and full configurations, respectively.

For the blind explanation review ($N = 100$ clips), Grounded Cue Precision is the proportion of cited cue statements judged observable in the source clip. Unsupported/Hallucinated Cue Rate is the proportion judged unsupported or contradicted. In a corpus-level text scan, exact category terms occurred in 6.8% of transcript records and near-synonymous phrases in 11.4%; masking explicit lexical cues retained 88.2% accuracy.

Supplementary Reporting Tables

The public metadata inventory contains 3960 rows, while the quality-controlled benchmark used in the experiments contains 3958 clips, including 3166 training, 397 validation, and 395 test clips. Table S3 reports the category-wise distribution reconstructed from the public inventory with the same source/scene grouping rule and seed 42.

Table S3: Category-wise train/validation/test distribution in the released metadata using source/scene grouping and seed 42 ($n = 3960$).

Category	Train	Validation	Test	Total
Radio communication and report	191	34	21	246
Tactical maneuver and G-load	138	16	15	169
Pre-flight inspection and preparation	121	12	18	151
Route cruise and formation	101	14	10	125
Emergency fault handling	48	9	14	71
Approach, landing, and carrier recovery	50	9	6	65
Weapon fire-control and strike	47	8	9	64
Ground taxi and takeoff	30	0	6	36
Tactical formation and cruise	240	31	27	298
High-maneuver flight and penetration	146	21	17	184
Takeoff and climb	135	18	11	164
Approach, landing, and arresting recovery	102	14	12	128
Weapon delivery and defensive countermeasure	64	10	14	88
Helicopter landing and special operations	20	0	1	21
Tactical briefing and deployment	642	86	90	818
Radar situation monitoring	244	34	32	310
Tower takeoff and landing control	224	16	31	271
Pre-flight servicing and boarding	195	15	22	232
Tactical debrief and performance review	84	13	8	105
Carrier-deck special support	81	8	4	93
Readiness-level elevation and assembly	74	5	7	86
Weapon loading and maintenance	65	6	6	77
Special-situation handling and emergency intervention	54	9	5	68
Weather assessment and decision	31	3	4	38
Ground taxi and marshalling	27	3	4	34
Electronic-warfare command	14	2	2	18
**Total**	**3168**	**396**	**396**	**3960**

Table S4: Per-category Precision, Recall, and F1 for the full model (MDEP–PPVG), computed from one-versus-rest predictions on the official test split ($n = 395$).

Category	Precision	Recall	F1
Radio communication and report	91.3	87.5	89.4
Tactical maneuver and G-load	88.9	88.2	88.5
Pre-flight inspection and preparation	93.3	86.7	89.8
Route cruise and formation	90.9	83.3	86.9
Emergency fault handling	87.5	100.0	93.3
Approach, landing, and carrier recovery	85.7	100.0	92.3
Weapon fire-control and strike	95.1	93.3	94.2
Ground taxi and takeoff	100.0	66.7	80.0
Tactical formation and cruise	93.1	90.0	91.5
High-maneuver flight and penetration	89.5	94.4	91.9
Takeoff and climb	93.8	93.8	93.8
Approach, landing, and arresting recovery	92.3	92.3	92.3
Weapon delivery and defensive countermeasure	88.9	88.9	88.9
Helicopter landing and special operations	100.0	100.0	100.0
Tactical briefing and deployment	89.2	91.4	90.3
Radar situation monitoring	79.2	78.0	78.6
Tower takeoff and landing control	88.5	85.2	86.8
Pre-flight servicing and boarding	91.3	91.3	91.3
Tactical debrief and performance review	90.0	80.0	84.7
Carrier-deck special support	88.9	88.9	88.9
Readiness-level elevation and assembly	87.5	87.5	87.5
Weapon loading and maintenance	85.7	85.7	85.7
Special-situation handling and emergency intervention	85.7	85.7	85.7
Weather assessment and decision	80.0	75.0	77.4
Ground taxi and marshalling	80.0	66.7	72.7
Electronic-warfare command	100.0	50.0	66.7

Table S5: Annotation review and provenance statistics.

**Analysis**	**Sample coverage**	**Agreement/result**	**Modification rate**
Intent-label review	15.2% ($n = 602$)	82.4%	17.6%
Cue-grounded explanation review	15.2% ($n = 602$)	78.6%	21.4%
Exact category-term scan in text	100.0% ($n = 3958$)	6.8% occurrence	n/a
Near-synonym scan in text	100.0% ($n = 3958$)	11.4% occurrence	n/a

Archived Research Release and Reuse Conditions

The current public Zenodo record contains the metadata CSV and its source titles, clip identifiers, segment timestamps when applicable, major perspective categories, fine-grained intent categories, transcripts where available, cue-grounded explanations, and annotation text. The record is available through the Zenodo DOI. The revision materials provide the deterministic grouping and split columns described above.

The Zenodo archive provides source titles, segment timestamps, annotations, and metadata for research reuse and source-segment retrieval. The underlying third-party video works remain with their original rights holders.
